# Immunosuppressive role of fibrinogen-like protein 2 (FGL2) in CD8^+^regulatory T cells-mediated long-term graft survival

**DOI:** 10.1186/1479-5876-9-S2-O5

**Published:** 2011-11-23

**Authors:** Séverine Bézie, Séverine Ménoret, Laurent Tesson, Xian-Liang Li, Claire Usal, Ignacio Anegon, Lise Caron

**Affiliations:** 1 INSERM UMR 643, Nantes, France; 2 Plateform Transgenic Rats Nantes IBiSA-CNRS, Nantes, France

## Background

We have previously reported that, in a model of cardiac allograft in rat, blockade of CD40-CD40L interaction induces long-term graft survival mediated by CD8^+^CD45RC^low^ regulatory T cells (Tregs) [1]. Transcriptomic comparison of Tregs from AdCD40Ig-treated vs naïve rats highlighted the overexpression of FGL2 whose immunoregulatory properties are little known [2].

## Material and methods

A lewis 1W rat heart is grafted in a MHC-mismatched Lewis 1A rat and infected with 2.10^10^pi of adenovirus recombinant for CD40Ig molecule (AdCD40Ig) the day of the graft. Tregs, effector CD4^+^CD25^-^ T lymphocytes (TL), and plasmacytoïde dendritic cells (pDC) from spleen are sorted by FACS Aria for *in vitro* tests. For *in vivo* studies, 4,5.10^11^vg of FGL2-recombinant adenovirus associated virus (AAVFGL2) are intramuscularly or intravenously injected in recipients 30 days before the graft. Splenocytes are transferred to sublethaly irradiated rats by *i.v* injection the day before the graft.

## Results

We confirmed FGL2-overexpression in splenic Tregs and in the graft of AdCD40Ig-treated vs non-treated and naïve rats, at mRNA and protein level. FGL2 involvement in Tregs immunosuppressive function was proved by *in vitro* and *in vivo* experiments. Indeed, Tregs from AdCD40Ig-treated rats inhibit TL proliferation in response to allogeneic pDC. This inhibition is abrogated by FGL2-blocking antibodies [3] and can be mimicked by FGL2 protein alone. Moreover, AAV-mediated FGL2 overexpression in rat prolongs graft survival with a median of 18.5 days vs 11 days for controls by i.m injection and survival is improved when i.v injected. Furthermore, adoptive transfer of splenocytes from an AAVFGL2-treated tolerant rat, to irradiated rats, transmits long-term graft survival iteratively.

## Conclusions

This is the first demonstration that the immunosuppressive molecule FGL2 is able to induce a long-term graft survival and that this tolerance is active and transferable by splenocytes. Work is under progress to identify the population responsible for this infectious tolerance.

## References

[B1] Guillonneau C Hill M Hubert FX Chiffoleau E Hervé C Li XL CD40Ig treatment results in allograft acceptance mediated by CD8CD45RC T cells, IFN-gamma, and indoleamine 2,3-dioxygenase J Clin Invest 2007 117 1096 1106 10.1172/JCI28801 17404623 PMC1839240

[B2] Liu H Shalev I The FGL2-FcgammaRIIB pathway: a novel mechanism leading to immunosuppression Eur J Immunol 2008 38 Suppl 11 3114 3126 18991288 10.1002/eji.200838338

[B3] Li XL Menoret S Mechanism and localization of CD8 regulatory T cells in a heart transplant model of tolerance J Immunol 2010 185 Suppl 2 823 833 20543104 10.4049/jimmunol.1000120

